# Two-dimensional isobutyl acetate production pathways to improve carbon yield

**DOI:** 10.1038/ncomms8488

**Published:** 2015-06-25

**Authors:** Yohei Tashiro, Shuchi H. Desai, Shota Atsumi

**Affiliations:** 1 Department of Chemistry, University of California, Davis, One Shields Avenue, Davis, California 95616, USA; 2 Microbiology Graduate Group, University of California, Davis, One Shields Avenue, Davis, California 95616, USA

## Abstract

For an economically competitive biological process, achieving high carbon yield of a target chemical is crucial. In biochemical production, pyruvate and acetyl-CoA are primary building blocks. When sugar is used as the sole biosynthetic substrate, acetyl-CoA is commonly generated by pyruvate decarboxylation. However, pyruvate decarboxylation during acetyl-CoA formation limits the theoretical maximum carbon yield (TMCY) by releasing carbon, and in some cases also leads to redox imbalance. To avoid these problems, we describe here the construction of a metabolic pathway that simultaneously utilizes glucose and acetate. Acetate is utilized to produce acetyl-CoA without carbon loss or redox imbalance. We demonstrate the utility of this approach for isobutyl acetate (IBA) production, wherein IBA production with glucose and acetate achieves a higher carbon yield than with either sole carbon source. These results highlight the potential for this multiple carbon source approach to improve the TMCY and balance redox in biosynthetic pathways.

## Abstract (web summary)

Achieving high carbon yields is crucial for biotechnological production of metabolites in engineered microorganisms. Here, Tashiro *et al*. generate *E. coli* strains that produce acetyl-CoA and a derived metabolite (isobutyl acetate) in the absence of pyruvate decarboxylation, leading to increased carbon yields.

## Graphical abstract

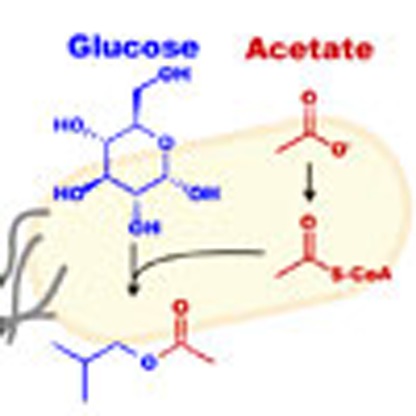

Biochemical production from biomass, a renewable resource synthesized from CO_2_ and sunlight, may contribute to a carbon-neutral society^1^. Increased understanding of cellular systems has enabled the re-design of metabolism^2^,^3^,^4^, and as a result microorganisms have been engineered to produce a range of fuels, chemicals and pharmaceuticals from biomass^5^,^6^,^7^,^8^,^9^,^10^,^11^,^12^. The main constraint in large-scale production has been in achieving efficiency that would allow economic feasibility.

The conversion ratio of an input carbon source to a target chemical is represented as carbon yield. A typical production system loses >30% of carbon during the production process^13^,^14^,^15^. For example, the well-established ethanol system produces 2 mol of ethanol and CO_2_ from 1 mol of glucose, which corresponds to a theoretical maximum carbon yield (TMCY) of 67% (4/6). High TMCY is required to achieve an economically competitive process.

One method to improve the TMCY is to avoid carbon loss by utilizing a carboxylation in which a carboxylic acid or CO_2_ is utilized^16^,^17^. Succinate production from glycerol via carboxylation achieved a TMCY of 133% (refs 18, 19) and the experimental carbon yield was 120% (ref. 20). However, carboxylation is limited to particular metabolic pathways, is not feasible for a wide range of chemicals, incurs high energetic costs, and is therefore often avoided.

Another method to improve the TMCY is to avoid decarboxylation. In cellular metabolism, acetyl-CoA is a building block for various metabolites such as amino acids, lipids and alcohols^21^. When sugars (that is, glucose) are used as starting substrates, acetyl-CoA is generated from pyruvate with carbon loss (Fig. 1a)^22^, reducing the TMCY in an acetyl-CoA-dependent pathway^23^. If acetyl-CoA can be generated without carbon loss, the TMCY would be enhanced for every acetyl-CoA-dependent pathway. Non-oxidative glycolysis (NOG) has been developed to produce acetyl-CoA from sugars without losing carbon^23^. Carbon yield from acetate as a substrate via the NOG reached 88% versus 67% via glycolysis. This work demonstrated that avoiding pyruvate decarboxylation is a useful approach to improve carbon yield. However, the NOG does not generate redox energy and therefore it is not directly applicable for production of high potential energy compounds such as alcohols or esters that depend on redox energy for their synthesis.

To achieve the TMCY, cofactor formation and consumption must be balanced because cofactor imbalance stagnates metabolic flow and decreases yield^3^. Cofactor balance is essential for chemical production in anaerobic conditions, a condition more economically feasible due to generally higher TMCY, and lower operation cost than aerobic production^24^,^25^. For instance, the isobutanol pathway first designed in *Escherichia coli*^26^ is redox imbalanced in anaerobic conditions. This was resolved by constructing a NADH-dependent isobutanol pathway^25^, which allowed 100% TMCY of isobutanol from glucose.

The goal of the present study was to achieve cofactor balance and decrease carbon loss to achieve an improved TMCY for target chemical production. Here we present a novel approach to improve the TMCY of isobutyl acetate (IBA), a chemical used as a flavouring agent, solvent and fuel^27^. IBA production in *E. coli* (Fig. 1a)^28^ is synthesized from isobutanol and acetyl-CoA by alcohol-*O*-acetyl transferase. Using glucose as the sole carbon source, IBA TMCY was 67%. In addition, two NADHs are in excess to produce one IBA from glucose. To simultaneously solve carbon loss and cofactor imbalance, we designed a novel pathway to produce the isobutanol and acetyl-CoA moieties from glucose and acetate, respectively (Fig. 1).

We construct two independent pathways to produce isobutanol from glucose, and acetyl-CoA from acetate, and then combine these pathways for IBA production. Acetate serves as the carbon source for acetyl-CoA, and is converted through an acetate-assimilating pathway without carbon loss or redox cofactor imbalance. Acetate is an inexpensive substrate and abundant in waste water at substantial concentrations^29^ that must be removed from waste streams^30^. This suggests that the production costs of the proposed system may be lower than those using glucose alone. To ensure that the only route to produce acetyl-CoA was from acetate, we remove the genes for native conversion of pyruvate to acetyl-CoA. In this strain, glycolysis and tricarboxylic acid cycle/fatty acid biosynthesis are completely divided. This novel redox balanced pathway increases the TMCY of IBA production from 67 to 75%, and increases the achieved carbon yield from 35 to 59%. Our use of multiple carbon sources and a divided metabolism could be applied to enhance and achieve the TMCY production for a range of biochemicals.

## Results

### Pathway design using two carbon sources

**Figure 1:**
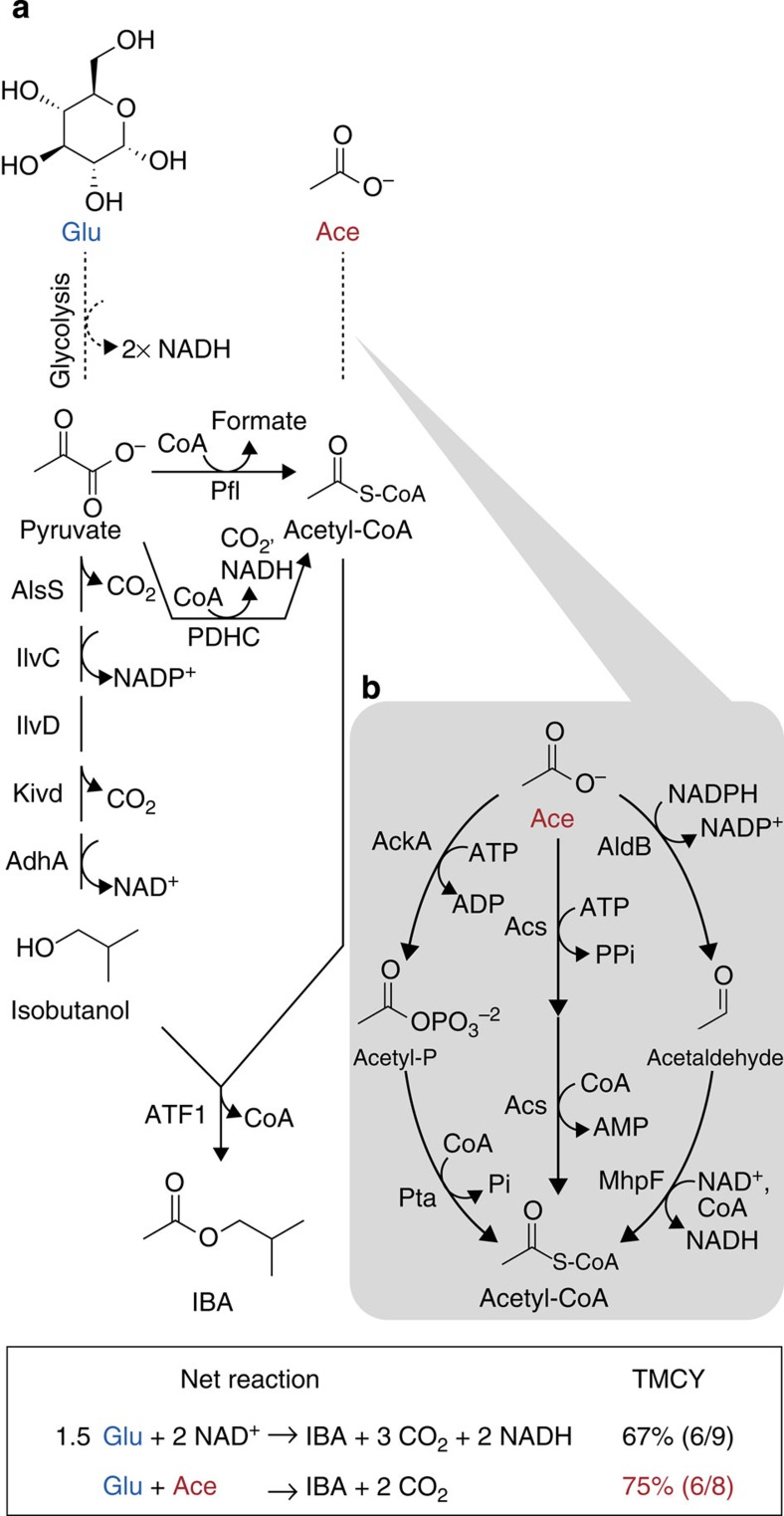
Pathway design for IBA synthesis. (**a**) IBA pathway from glucose and acetate. (**b**) Acetate-assimilating pathways from *E. coli*. Ace, acetate; acetyl-P, acetyl phosphate; AdhA, alcohol dehydrogenase from *L. lactis*; AlsS, acetolactate synthase from *Bacillus subtilis*; ATF1, alcohol-*O*-acetyl transferase from *Saccharomyces cerevisiae*; Glu, D-glucose; IBA; isobutyl acetate; IlvC, 2-hydroxy-3-ketol-acid reductoisomerase from *E. coli*; IlvD, dihydroxy-acid hydratase from *E. coli*; Kivd, 2-keto acid decarboxylase from *Lactococcus lactis*.

Previously, an IBA pathway was designed in *E. coli* using glucose as the sole carbon source^28^. IBA production requires two moieties: isobutanol and acetyl-CoA; isobutanol can be effectively synthesized from two molecules of pyruvate with the aid of five enzymes (AlsS, IlvCD, Kivd and AdhA) in *E. coli* (Fig. 1)^26^. Acetyl-CoA, the second moiety, is also generated from pyruvate when glucose is used as a sole carbon source. *E. coli* has two main routes for acetyl-CoA formation, which are catalysed by pyruvate formate lyase (Pfl) or pyruvate dehydrogenase complex (PDHC)^22^. These enzymes have high activity in *E. coli*, however, these reactions cause carbon loss by releasing formate or CO_2_. *E. coli* strain JCL260 (ref. 26), which is the host for IBA production (Supplementary Table 1) does not contain *pflB* encoding Pfl. In JCL260, PDHC is the only enzyme present to generate acetyl-CoA from pyruvate directly. NADH generated by PDHC during acetyl-CoA formation causes a redox imbalance in the IBA synthesis pathway (Fig. 1a). Therefore, the TMCY for IBA when glucose is used as a sole carbon source is 67%.

To improve TMCY for IBA production, the IBA synthesis pathway was split into two modules in which acetate was used for acetyl-CoA generation and glucose was converted solely into isobutanol (Fig. 1a). *E. coli* can naturally utilize acetate as a carbon source, however, the efficiency is very low. To enhance acetate assimilation efficiency, several acetate-assimilating pathways were identified (Fig. 1b). These pathways are balanced in redox and they do not cause carbon loss. Co-utilization of glucose and acetate as a carbon source increases carbon yield of IBA up to 75% and balances redox in the IBA pathway. All strains used in this study are described in Table 1.

### Screening acetate-assimilating pathway

**Figure 2:**
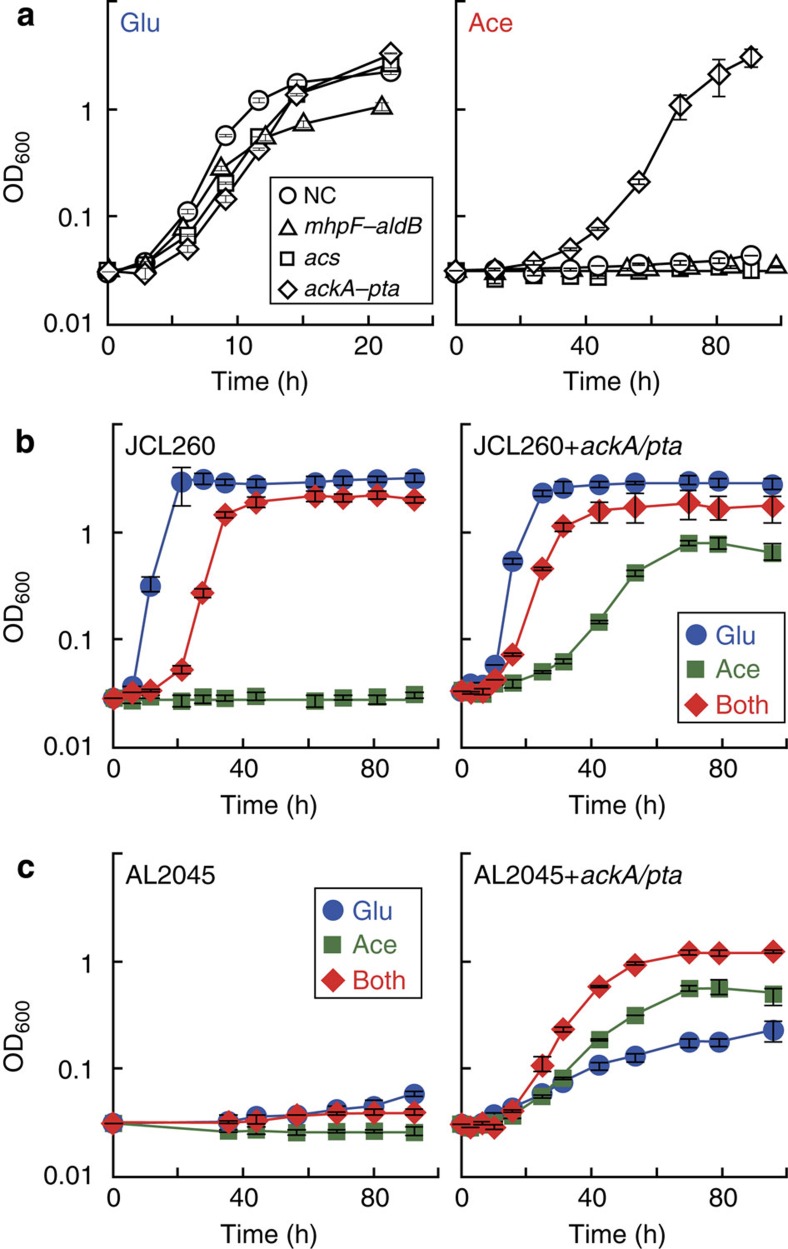
Acetate-assimilating pathway construction. (**a**) Three different acetate-assimilating pathways were constructed in JCL260 (strains 1 (diamond), 2 (square), 3 (triangle) or 4 (circle); Table 1) and screened in M9 minimal media with 10 g l^−1^ acetate as a sole carbon source; NC represents negative control using strain 4. Growth of JCL260 (**b**) and AL2045 (**c**) on acetate and glucose. The right panel shows growth of strains 1 (**b**) and 5 (**c**) while the left panel depicts growth of strains 4 (**b**) and 6 (**c**) Error bars indicate s.d. (*n*=3).

Three acetate-assimilating pathways were constructed (Fig. 1b). In the acetate kinase–phosphotransacetylase (AckA–Pta) pathway^31^, acetate is phosphorylated by AckA using ATP to produce acetyl phosphate, which is then converted into acetyl-CoA by Pta. The acetyl-CoA synthetase (Acs) pathway consists of just one enzyme: Acs, which requires ATP and proceeds through acetyladenylate as an intermediate^31^. Last is the AldB–MhpF pathway^32^,^33^, which potentially could help improve TMCY because this pathway does not utilize ATP. Both AldB and MhpF are known as acetaldehyde dehydrogenase, but MhpF is CoA-dependent. To identify which pathway would allow the most acetate consumption, genes for the different acetate-assimilating pathways were individually introduced in JCL260 (strains 1, 2 and 3, respectively, Table 1) and the growth of the strains was compared with 10 g l^−1^ glucose or 10 g l^−1^ acetate as a sole carbon source in micro-aerobic conditions (Fig. 2a).

Expressing genes in each pathway did not have a significant effect on the growth of JCL260 in M9 minimal media with 10 g l^−1^ glucose, indicating that these enzymes are not toxic when glucose is used as a sole carbon source (Fig. 2a, left). Interestingly, only the AckA–Pta pathway enabled JCL260 to grow with 10 g l^−1^ acetate (Fig. 2a, right). The AckA–Pta pathway is reversible, therefore, this pathway is also known as an acetate-generating pathway rather than an acetate-assimilating pathway^34^. However, at high acetate concentrations, the AckA–Pta pathway has greater activity for acetate assimilation than the Acs pathway^31^. AckA binds acetate poorly (*K*_m_=7 mM)^35^, while Acs binds with much higher affinity (*K*_m_=0.2 mM)^36^. However, in this study 10 g l^−1^ (0.17 M) acetate was used, which is much higher than the *K*_m_ value of AckA. Higher substrate concentration pushes the reaction forward towards the product. This result indicates that the AckA–Pta pathway is suitable for acetyl-CoA formation and subsequent IBA production.

### Effect of AckA–Pta pathway on *E. coli* growth

To prevent pyruvate decarboxylation, *aceEF* encoding PDHC was removed from JCL260, resulting in AL2045 (see Methods, Supplementary Table 1). AL2045 is not able to generate acetyl-CoA directly from pyruvate (Fig. 1a). The AckA–Pta pathway was introduced into JCL260 and AL2045 to observe the growth of these strains in M9 minimal media with 10 g l^−1^ of glucose, 10 g l^−1^ of acetate or both (Fig. 2b,c).

JCL260 with and without the AckA–pta pathway (strains 1 and 4, respectively, Table 1) had similar growth trends (Fig. 2b). These strains grew slower with glucose and acetate than with only glucose. These results indicated that the AckA–Pta pathway did not have an effect on growth. The slow growth may be attributed to reduced glycolysis activity in high acetate concentrations^37^,^38^. AL2045 without the AckA–Pta pathway (strain 6, Table 1) was not able to grow in these three culture conditions (Fig. 2c, left) because strain 6 is unable to generate acetyl-CoA from pyruvate. On the other hand, AL2045 harbouring the AckA–Pta pathway (strain 5) grows in all the conditions tested (Fig. 2c, right). Strain 5 lacks a direct route from pyruvate to acetyl-CoA, however, this strain still has an indirect route to generate acetyl-CoA via acetate. Pyruvate oxidase generates acetate from pyruvate, acetate can then be converted to acetyl-CoA through the AckA–Pta pathway. In this way, strain 5 was able to grow with glucose as a sole carbon source. Strain 5 grew faster in the presence of both acetate and glucose than either sole carbon source (Fig. 2c, right). *E. coli* K12 has the ability to convert acetate to acetyl-CoA and synthesize important metabolites, such as pyruvate, lipids and amino acids from acetyl-CoA via the tricarboxylic acid cycle^39^. This synergetic effect of glucose and acetate implies that to produce pyruvate and acetyl-CoA from glucose and acetate, respectively, is more efficient than solely from either carbon source.

### IBA production on various carbon source conditions

**Figure 3:**
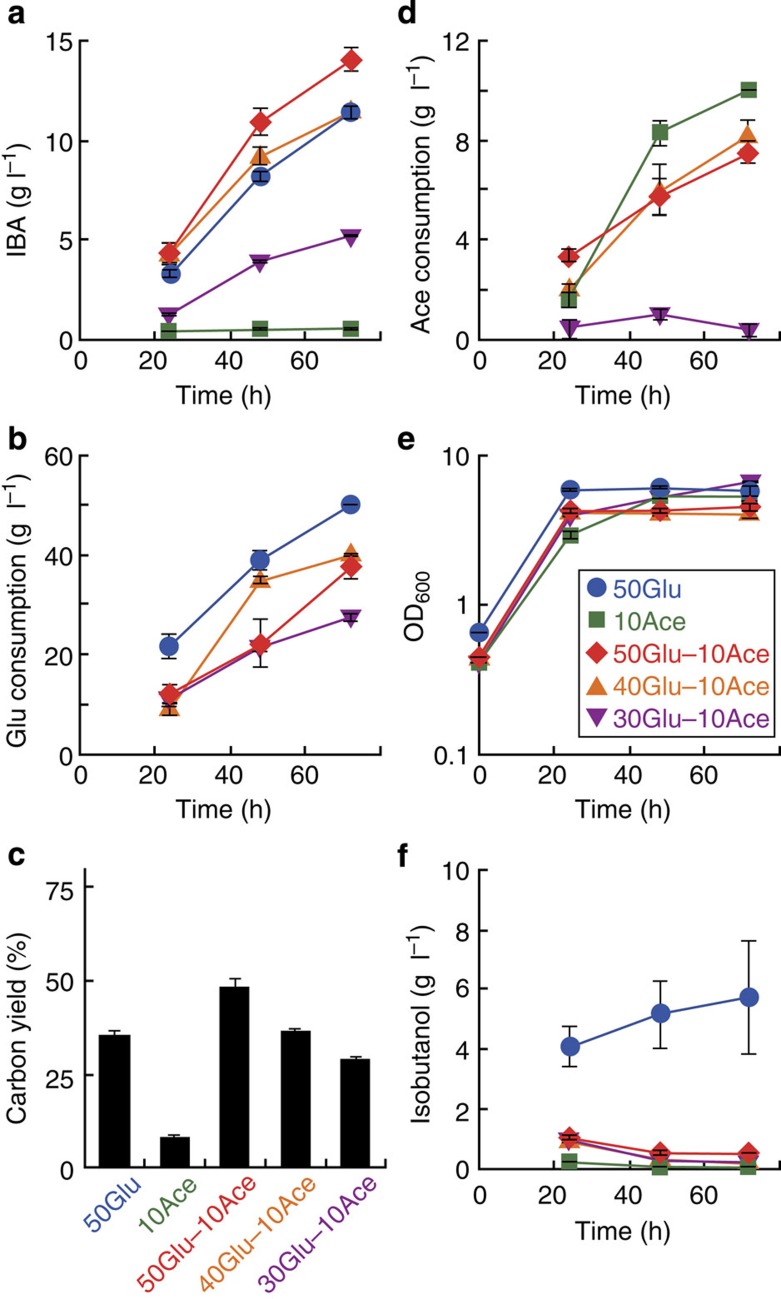
IBA production with glucose and acetate in strain 7. Strain 7 (JCL260 harbouring IBA production and acetate-assimilating pathways, Table 1) was grown in M9P media with 50 g l^−1^ glucose (50Glu), 10 g l^−1^ acetate (10Ace) or both (50Glu–10Ace); or 40 g l^−1^ glucose and 10 g l^−1^ acetate (40Glu–10Ace); or 30 g l^−1^ glucose and 10 g l^−1^ acetate (30Glu–10Ace). IBA concentration (**a**), isobutanol concentration (**f**), consumed glucose (**b**), consumed acetate (**d**) and cell growth (**e**) were monitored during the experiment. Carbon yield of IBA was calculated at 72 h (**c**) Error bars indicate s.d. (*n*=3).

On identification that Ack–Pta pathway was the best acetate-assimilating pathway in the three tested pathways, it was combined with the IBA synthesis pathway in JCL260 and AL2045 (strains 7 and 8, respectively, Table 1). To identify a good condition for IBA production varying concentrations of glucose and acetate were investigated. The strains were grown in M9P (modified M9 minimal media see Methods) with glucose, acetate or both for 3 days and IBA production was monitored (Fig. 3, Supplementary Fig. 2). The carbon yield of IBA (CY_IBA_) is defined with the following formula:


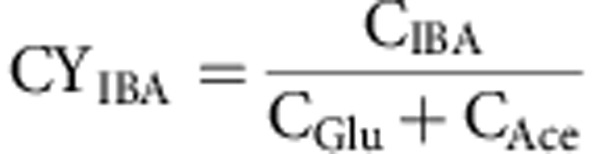


where C_IBA_, C_Glu_ and C_Ace_ is the number of carbons molecules (# of carbons per molecule × concentration (M)) of IBA, fed glucose and fed acetate, respectively.

After 72 h, strain 7 produced 11.4 g l^−1^ IBA from 50 g l^−1^ glucose as a sole carbon source (Fig. 3a,b) and the CY_IBA_ was 35.3% after 72 h (Fig. 3c). Conversely, when 10 g l^−1^ acetate was used as a sole carbon source, all of the acetate was consumed in 72 h (Fig. 3d), <1 g l^−1^ IBA was produced and the CY_IBA_ was 8.2% (Fig. 3a,c,d). The final cell density of strain 7 grown with acetate was similar to that with glucose (Fig. 3e). These results indicate that the low IBA production from acetate is because there is low pyruvate availability for isobutanol production. In addition, when strain 7 was grown on glucose, 5.7 g l^−1^ of isobutanol remained in the media and was not converted into IBA (Fig. 3f). Since, IBA production requires the condensation of isobutanol and acetyl-CoA, excess isobutanol suggested that acetyl-CoA generation was rate limiting. Additional acetate as an acetyl-CoA source would improve IBA titre and CY_IBA_.

For further improvement of IBA production, strain 7 was grown in various glucose concentrations (50, 40 or 30 g l^−1^) and 10 g l^−1^ acetate were simultaneously used as carbon sources. Acetate concentration was fixed at 10 g l^−1^ in this experiment because >10 g l^−1^ acetate inhibits *E. coli* growth (Supplementary Fig. 1). On assessing IBA titre and CY_IBA_ from different glucose concentrations it appeared that these factors were dependent on glucose concentration (Fig. 3a,c). Media containing, 50 g l^−1^ glucose and 10 g l^−1^ acetate allowed strain 7 to produce 13.9 g l^−1^ IBA after 72 h and the CY_IBA_ reached 47.8%. On the other hand, isobutanol titre was significantly reduced to 0.6 g l^−1^ (Fig. 3f). This indicates that acetyl-CoA became more abundant by fed acetate and acetylation of isobutanol was highly active. In spite of high IBA titre and CY_IBA_, the glucose consumption (37.5 g l^−1^) and acetate consumption (7.5 g l^−1^) were low compared with sole consumption of either glucose or acetate (Fig. 3b,d). This result shows that co-utilization of glucose and acetate led to more efficient IBA production. In theory, 1 mol of IBA is synthesized from 1 mol of glucose and 1 mol of acetate (Fig. 1a). Media containing 30 g l^−1^ glucose and 10 g l^−1^ acetate represent the 1 to 1 M ratio of these carbon sources and would be an ideal media recipe if these carbon sources are consumed in equivalent molar ratios. However, IBA production was relatively low in this media condition (5.2 g l^−1^ IBA and 29.2% CY_IBA_) (Fig. 3a,c). In addition, very little to no acetate was consumed (Fig. 3d). Low IBA production may be due to a complex relationship between glucose and acetate in *E. coli* metabolism and regulation. Omics analysis may help to elucidate this observation in the future.

In strain 8, IBA was produced only when the strain was grown in M9P with both glucose and acetate (Supplementary Fig. 2). When glucose or acetate was used as the sole carbon source, this strain produced 3.5 or 0.3 g l^−1^ isobutanol, respectively (Supplementary Fig. 2f). Since strain 8 is disabled to generate acetyl-CoA from glucose, isobutanol was not converted to IBA. When both required carbon sources are present, 5.9 g l^−1^ IBA was produced and CY_IBA_ was 25.4% from 50 g l^−1^ glucose and 10 g l^−1^ acetate (Supplementary Fig. 2a,c). There is a large difference between 50 g l^−1^ glucose and <50 g l^−1^ glucose in IBA titre and CY_IBA_.

To improve IBA production by using AL2045 (JCL260 Δ*aceEF*), the regulation for the isobutanol synthesis pathway and acetate-assimilating pathway were split, and we attempted to optimize expression levels of these pathways. Originally, isopropyl β-D-1-thiogalactopyranoside (IPTG)-inducible promoter, P_L_lacO_1_ (ref. 40), was used for both pathways. *ackA* and *pta* were put under the control of anhydrotetracycline (aTc)-inducible promoter, P_L_tetO_1_ (ref. 40). In the new construct, the genes in the IBA synthesis pathway, those required for isobutanol synthesis and the acetate-assimilating pathways can be individually induced by IPTG and aTc, respectively.

AL2045 harbouring the isobutanol and acetate-assimilating pathways under the control of P_L_tetO_1_ (strain 9, Table 1) was grown in M9P including 50 g l^−1^ glucose and 10 g l^−1^ acetate. The AckA–Pta pathway was activated by different aTc concentrations and the resulting IBA production was monitored (Supplementary Fig. 3). Final cell density was dependent on aTc concentration (Supplementary Fig. 3e). Isobutanol was detected in the absence of aTc but not in the presence of aTc (Supplementary Fig. 3f). These results show that higher aTc concentrations allowed more available acetyl-CoA in this strain. However, IBA titre and CY_IBA_ were not simply increasing with greater aTc concentration. In spite that 2.1 g l^−1^ isobutanol remained in the culture without aTc, it resulted in the best IBA production (4.3 g l^−1^ and CY_IBA_: 21.2%). Surprisingly, orthogonal induction of the two pathways required for IBA production resulted in lower IBA titres than inducing both pathways with a single inducer (Supplementary Figs 2 and 3). Varying expression levels of the acetate-assimilating pathways most likely altered acetyl-CoA and its derivatives, such as acetyl-P, availability in the cell and therefore impacted the gene expression profile^37^,^41^. For example, fatty acid production is regulated by acetyl-CoA concentration in *E. coli*^42^. Zhang *et al*.^43^ designed a dynamic regulator system based on acetyl-CoA availability for diesel ester production and their system showed better production than using a constitutive promoter. Such a dynamic regulation system would be useful for IBA production.

### Isotope tracing of IBA synthesis pathway

**Figure 4:**
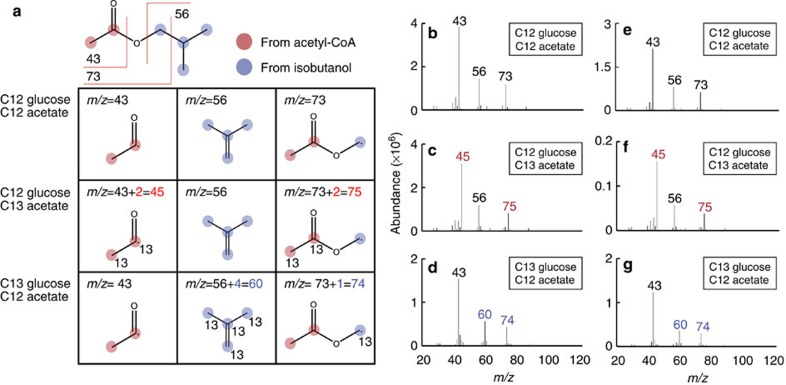
Isotope tracing of the IBA synthesis pathway using ^13^C glucose and ^13^C acetate. (**a**) IBA structure and predicted MS fragmentation pattern. The location of predicted ^13^C is represented by the number 13 adjacent to the carbon molecules. IBA was produced using unlabelled substrate (**b**,**e**), ^12^C glucose and ^13^C acetate (**c**,**f**) or ^13^C glucose and ^12^C acetate (**d**,**g**) in strain 7 (**b**–**d**) or 8 (**e**–**g**).

To investigate that both glucose and acetate are contributing towards IBA production, isotopic tracing analysis using ^13^C glucose or ^13^C acetate was performed. Strains 7 and 8 (Table 1) were grown with several combinations of carbon sources for IBA production and analysed by capillary gas chromatography mass spectrometry (GC–MS; (Fig. 4).

In Fig. 4 carbon atoms in IBA originating from acetyl-CoA or isobutanol are emphasized in red and blue, respectively. Three major IBA fragments (*m*/*z*=43, 56 and 73) were observed in the MS analysis for IBA produced from ^12^C glucose and ^12^C acetate in strains 7 and 8 as was predicted by the NIST MS 2.0 library^44^ (Fig. 4). The fragment C_2_H_3_O· corresponds to a *m*/*z=*43, C_4_H_8_· fragment arises from a McLafferty rearrangement^45^ giving a *m*/*z=*56, and *m*/*z=*73 corresponds to a C_3_H_5_O_2_· fragment.

Next, the IBA producing strains were fed with ^13^C acetate instead of ^12^C acetate (Fig. 4c,f). Two fragments at *m*/*z=*43 and 73 were not detected and instead peaks at 45 and 75 were observed; *m*/*z=*43 and 73 fragments from IBA contain two carbon atoms originating from acetyl-CoA (Fig. 4a). Observation of fragments 2 units heavier than with unlabeled substrate (*m*/*z=*43 to 45 and 73 to 75), demonstrates that the acetyl group in IBA originated from the labelled acetate. No observation of fragments with *m*/*z=*43 and 73 indicated that almost all of the IBA was produced with acetyl-CoA from acetate as a carbon source.

When ^13^C glucose was used as an isobutanol source with ^12^C acetate, different mass to charge ratios were observed (Fig. 4d,g). Here, *m*/*z=*56 and 73 observed with unlabelled carbon were shifted to 60 and 74, respectively. A fragment with *m*/*z*=56 contains four carbon atoms originating from isobutanol while the fragment with *m*/*z*=73 contains only one carbon atom from isobutanol (Fig. 4a). Isobutanol is synthesized from glucose (Fig. 1) and the observed peak shifts with heavy glucose were consistent with predicted structural analysis. The lack of detectable *m*/*z*=56 and 73 and detection of shifted peaks supports that the isobutanol moiety in IBA is from glucose and not acetate.

Interestingly, strains 7 and 8 showed similar MS patterns, despite the fact that strain 7 has a PDHC. A PDHC would allow direct conversion of pyruvate (from glucose) to acetyl-CoA. However, based on the fragmentation patterns from strain 7 it appears that acetyl-CoA is mainly generated from acetate instead of pyruvate. When acetate is used as a sole carbon source in *E. coli*, it is known that PDHC expression is reduced^37^. In this experiment, acetate was not used as a sole carbon source, however, it is still possible that PDHC expression was reduced in the cell. Another possibility for decreased PDHC activity is that pyruvate concentration decreased in the cell. Pyruvate concentration >15 mM activates PDHC expression via pyruvate-dependent transcriptional regulator, PdhR^46^. AlsS in the isobutanol pathway has a strong activity and high affinity (*K*_m_=13.6 mM) for pyruvate^47^ and could reduce pyruvate concentration in the cell. In addition, during IBA production, using glucose as a sole carbon source, a large amount of isobutanol was not converted to IBA and remained in the culture suggesting a low amount of acetyl-CoA (Fig. 3f). These facts suggest that PDHC activity was not high in strain 7.

### IBA production with acetate-feeding

**Figure 5:**
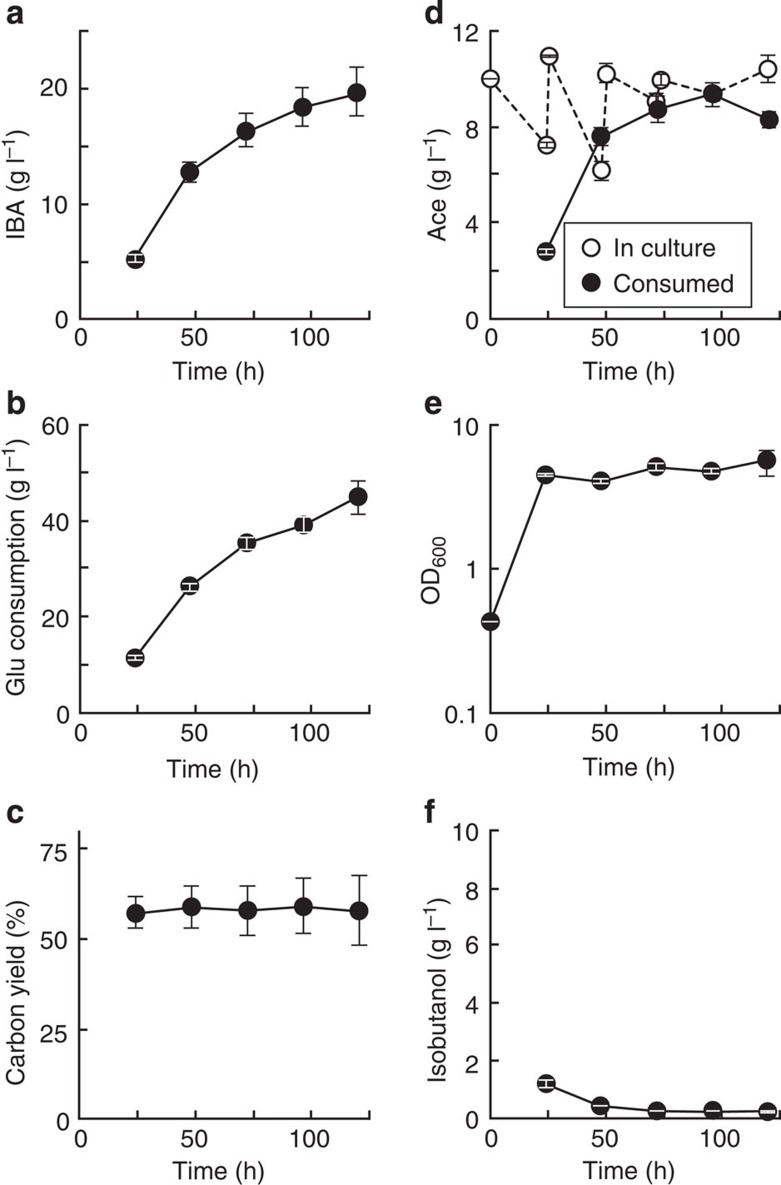
IBA production with acetate-feeding in strain 7. Strain 7 (JCL260 harbouring IBA production and acetate-assimilating pathways, Table 1) was grown in M9P media with 50 g l^−1^ glucose and 10 g l^−1^ acetate, where acetate was fed daily. IBA concentration (**a**), isobutanol concentration (**f**), consumed glucose (**b**), consumed acetate (**d**) and cell density (**e**) were monitored during the experiment. Carbon yield of IBA was calculated during the entire experiment (**c**). Error bars indicate s.d. (*n*=3).

Strain 7 grown with 50 g l^−1^ glucose and 10 g l^−1^ acetate allowed for the best IBA titre in the tested conditions (Fig. 3). To convert all 50 g l^−1^ glucose (0.27 M) to IBA, 10 g l^−1^ acetate (0.17 M) is not molecularly sufficient. (Fig. 3a,b,d). Additional acetate would be required for IBA production, however, increasing acetate concentration in the media is unfavourable for *E. coli* growth (Supplementary Fig. 1). Therefore, in the following experiments, acetate concentration was maintained at 10 g l^−1^ by feeding acetate every 24 h and IBA production was monitored for 5 days (Fig. 5, Supplementary Figs 4 and 5).

During IBA production in strain 7, CY_IBA_ was 57–59% and the IBA titre was 19.7 g l^−1^ after 120 h of production (Fig. 5). Until 72 h, the molecular consumption ratio of glucose and acetate (1.3:1) was relatively close to 1:1, which is the ideal consumption ratio for IBA synthesis. Glucose consumption constantly increased over time in this experiment, however, acetate consumption stopped at ∼9 g l^−1^ at 96 h. Conversely, the strain produced ∼1 g l^−1^ acetate between 96 and 120 h.

The same IBA production test was performed in strains 8 and 9 (Supplementary Figs 4 and 5). In strain 8, the highest IBA titre was 7.1 g l^−1^ and the CY_IBA_ achieved was 49.9% after 120 h incubation (Supplementary Fig. 4a,c). The carbon source consumption ratio between glucose and acetate in strain 8 was closer to the ideal 1:1 ratio for IBA production during this experiment than in strain 7. However, the titre and CY_IBA_ in strain 8 were lower in comparison to strain 7. One issue using strain 8 is the low consumption of carbon sources (17.1 g l^−1^ glucose and 5.0 g l^−1^ acetate) compared with strain 7 (Supplementary Fig. 4b,d). More than half of the glucose was not consumed. Acetate consumption was stopped at 96 h and acetate was produced between 96 and 120 h. Conversely, with the aTc-inducible *ackA*–*pta* construct, both glucose and acetate were continuously consumed throughout production (Supplementary Fig. 5b,d). In strain 9, the IBA titre achieved 9.5 g l^−1^ at 120 h (Supplementary Fig. 5a), which was better than in strain 8 (Supplementary Fig. 4a). On the other hand, the CY_IBA_ was 33.9% on average (Supplementary Fig. 5c) and 3.0 g l^−1^ isobutanol was not converted to IBA (Supplementary Fig. 5f). Our results show that the expression levels of *ackA*–*pta* has an effect not only on acetate consumption, but also on glucose.

## Discussion

Methods to engineer *E. coli* for chemical production has been well established by previous studies^2^,^3^,^4^. In most biochemical production scenarios, sugar (that is, glucose, xylose, glycerol and so on) is used as a starting substrate because these are abundant in nature. Acetate is another carbon source candidate because it is also abundant in nature, especially, in agricultural and industrial waste water. However, the acetate-assimilating pathway in wild-type *E. coli* is not efficient compared with glycolysis. We constructed an efficient acetate-assimilating pathway (AckA–Pta pathway) in *E. coli* and demonstrated that the pathway is useful for *E. coli* growth and IBA production at high acetate concentrations. In this study, glucose and acetate were co-utilized for IBA production. This strategy increases the TMCY of IBA and balances redox in the pathway by using acetate as an acetyl-CoA source and drastically enhanced the IBA titre and the CY_IBA_ (Figs 3 and 5 and Supplementary Figs 2, 4 and 5). This successful IBA production emphasized that avoiding carbon loss and balancing redox in designed metabolic pathways is crucial for biochemical production. Anaerobic conditions and strict control of acetate will further increase the IBA titre and the CY_IBA_. Even though AckA–Pta was overexpressed in *E. coli*, acetate consumption rate in *E. coli* is still low compared with glycolysis (Fig. 5). To improve productivity of IBA, faster acetate consumption is preferred. In nature, some soil microorganisms have better ability to assimilate acetate than *E. coli*^48^. Enzymes from these organisms might have higher activity than those from *E. coli.* Their introduction in *E. coli* may improve this pathway and enhance productivity.

Acetyl-CoA is used as a building block for amino acid biosynthesis, fatty acid biosynthesis and terpenoid biosynthesis. These pathways contribute to desirable and valuable chemicals such as food, fuels and pharmaceuticals. In these pathways, carbon loss and excess redox energy are caused by pyruvate decarboxylation^23^,^49^,^50^. Using acetate as an acetyl-CoA source is applicable for these pathways and would increase the carbon yield of useful chemicals. Furthermore, acetate-to-acetyl-CoA pathways could be extended to higher chain acyl-CoA (that is, butyryl-CoA) without redox energy via the reverse β-oxidation pathway^51^,^52^. Usage of acetate as an acetyl-CoA source in the designed pathway provides another route to make biochemical production potentially feasible.

## Methods

### Reagents

All enzymes were purchased from New England Biolabs (NEB). All oligonucleotide synthesis and DNA sequencing were performed by Eurofins MWG Operon Inc. (Huntsville, AL). All chemicals for GC standards were purchased from Sigma-Aldrich. ^13^C labelled substrates (D-glucose (U-13C6, 99%) and sodium acetate (1,2-13C2, 99%)) were purchased from Cambridge Isotope Laboratories Inc. (Tewksbury, MA).

### Strains and plasmids

All strains, plasmids and oligonucleotides are listed in Supplementary Table 1 and Supplementary Table 2. JCL260 and AL2045 were used for screening acetate assimilation and IBA production. JCL260 was previously developed for isobutanol production^26^. AL2045 was constructed from JCL260 with a deletion in *aceEF*. The knockout was constructed by the Wanner method^53^ using primers YT706 and YT707 to make the gene disruption fragment cassette. The strain was verified by using primers YT712 and YT713.

The target gene(s) and vector fragments to construct plasmids were amplified with the pairs of primers from the templates listed in Supplementary Table 3. The resulting fragments were combined by sequence and ligation-independent cloning^54^. Plasmids were verified by digestion with restriction enzymes and sequencing.

### Culture conditions

Overnight cultures were grown in 5 ml Luria broth media containing appropriate antibiotics. Antibiotic concentrations were as follows: kanamycin (50 μg ml^−1^; IBI Scientific), ampicillin (200 μg ml^−1^; Fisher BioReagents), tetracycline (20 μg ml^−1^; Fisher BioReagents) and spectomycin (50 μg ml^−1^). IBA production was carried out with M9P medium, consisting of M9 medium (33.7 mM Na_2_HPO_4_, 22 mM KH_2_PO_4_, 8.55 mM NaCl, 9.35 mM NH_4_Cl, 1 mM MgSO_4_ and 0.1 mM CaCl_2_; BD Bacto); 5 g l^−1^ yeast extract (BD Bacto); 50 g l^−1^ glucose (Fisher BioReagents); and 1,000-fold dilution of A5 trace metal mix (2.86 g H_3_BO_3_ (Fisher Chemical), 1.81 g MnCl_2_·4H_2_O (Alfa Aesar), 0.079 g CuSO_4_·5H_2_O (Sigma-Aldrich) and 49.4 mg Co(NO_3_)_2_·6H_2_O (Sigma-Aldrich) per liter water). Optical densities (OD) were measured at 600 nm with a Synergy H1 hybrid plate reader (BioTek Instruments, Inc.).

### Screening acetate-assimilating pathway in *E. coli*

Overnight culture was grown in 3 ml Luria broth media. Cell culture was centrifuged at 3,500 *g* for 2 min and the cell pellet was re-suspended with fresh M9 minimal media without carbon source. The concentrated cells were inoculated at 1% into 5 ml of M9 minimal media, containing 10 g l^−1^ glucose or 10 g l^−1^ acetate as a sole carbon source, with 1 mM IPTG in 15-ml screw-cap test tube, at 37 °C and 250 r.p.m.

### IBA production

Overnight cultures were inoculated 1% in 70 ml of M9P with appropriate carbon source(s) in 250-ml baffled flask. Cells were grown to an OD_600_ of ∼0.4 at 37 °C, 250 r.p.m., followed by induction with 1 mM IPTG. Then, 20 ml of culture was transferred to a new 250-ml screw-cap flask and 20 ml of *n*-hexadecane (ACROS Organics) was added to each flask. The cultures were shifted to 30 °C, 250 r.p.m. for the duration of the experiment.

### GC sample preparation

Isobutanol and IBA were analysed by GC with a flame ionization detector (GC–FID). This protocol was based on previous report^28^. For isobutanol analysis, 2 ml of cell culture was centrifuged at 21,000 *g* for 10 min at 4 °C and 1 ml of the supernatant was analysed via GC–FID. For hexadecane layer-assisted IBA production, 2 ml from the hexadecane layer was centrifuged at 21,000 *g* for 3 min, subsequently 1 ml of the hexadecane fraction was taken for GC analysis.

### GC analysis for isobutanol and IBA

Concentrations of IBA and isobutanol were analysed by GC–FID. The GC system is a GC-2010 with an AOC-20 S auto sampler and AOC-20i auto injector (Shimadzu). The column used was a DB-FFAP capillary column (60-m length, 0.32-mm diameter and 1-μm film thickness; Agilent Technologies; Santa Clara, CA, USA). The GC oven temperature was held at 225 °C, and the FID was held at 330 °C. The injection volume was 0.5 μl, injected at a 15:1 split ratio. Helium was used as the carrier gas. 1-Pentanol was used as an internal standard. Retention times from samples were compared with external standards. All samples contained 100 mg l^−1^ of 1-pentanol which served as an internal standard.

### Glucose and acetate analysis by HPLC

To determine carbon yield to produce IBA, glucose and acetate concentrations were measured every 24 h by centrifuging culture fraction for 10 min at 20,000 *g*. The cell supernatant was measured using a 20A high-performance liquid chromatography (HPLC from Shimazu) equipped with a differential refractive detector 10A and an Aminex fast acid analysis column (Bio-Rad). The mobile phase was 5 mM of H_2_SO_4_, maintained at a flow rate of 0.6 ml min^−1^ at 65 °C for 12.5 min.

### C13 analysis

C13 analysis was performed using a GC 6890N Network system (Agilent) with a Factor Four Capillary CP8944 column (30-m length, 0.25-mm diameter and 0.25-μm film thickness) (Agilent). The GC oven temperature was held at 40 °C for 4 min and then was increased at a rate of 45 °C min^−1^ until 300 °C and held for 3 min. The injector temperature was held at 250 °C. The injection volume was 2 μl, injected at a 100:1 split ratio. Helium was used as the carrier gas. The MS is a 5,973 Network mass selective detector (Agilent). The ion source temperature was 200 °C, and the interface temperature was 250 °C. The solvent cut time was 3 min hexadecane. Detector voltage was −0.1 kV. The start *m*/*z* was 20, and the end *m*/*z* was 550.

## Additional information

**How to cite this article:** Yohei, T. *et al*. Two-dimensional isobutyl acetate production pathways to improve carbon yield. *Nat. Commun.* 6:7488 doi: 10.1038/ncomms8488 (2015).

## Supplementary Material

Supplementary Information Supplementary Figures 1-5, Supplementary Tables 1-3 and Supplementary References

## Figures and Tables

**Table 1:** List of strains used in this study.

Strain no.	Host	Plasmid	Plasmid contents
1	JCL260	pAL953	*P*_L_lacO_1_: *ackA*–*pta*
2	JCL260	pAL954	*P*_L_lacO_1_: *acs*
3	JCL260	pAL955	*P*_L_lacO_1_: *mhpF–aldB*
4	JCL260	pAL956	*P*_L_lacO_1_: *sfGFP*
5	AL2045	pAL953	*P*_L_lacO_1_: *ackA*–*pta*
6	AL2045	pAL956	*P*_L_lacO_1_: *sfGFP*
7	JCL260	pAL953pAL603pAL991	*P*_L_lacO_1_: *ackA*–*ptaP*_L_lacO_1_: *alsS–ilvCD*, *P*_L_lacO_1_: *kivd–adhAP*_L_lacO_1_: *ATF1*
8	AL2045	pAL953pAL603pAL991	*P*_L_lacO_1_: *ackA*–*ptaP*_L_lacO_1_: *alsS–ilvCD*, *P*_L_lacO_1_: *kivd–adhAP*_L_lacO_1_: *ATF1*
9	AL2045	pAL1022pAL603pAL991	*P*_L_tetO_1_: *ackA*–*ptaP*_L_lacO_1_: *alsS–ilvCD*, *P*_L_lacO_1_: *kivd–adhAP*_L_lacO_1_: *ATF1*
