# Supplementary material for: Two-dimensional isobutyl acetate production pathways to improve carbon yield
Source: Nat Commun. 2015 Jun 25;6:7488. doi: 10.1038/ncomms8488 (PMC4491173; doi:10.1038/ncomms8488)
Supplement: Supplementary Information — Supplementary Figures 1-5, Supplementary Tables 1-3 and Supplementary References [file ncomms8488-s1.pdf]

Supplementary Information

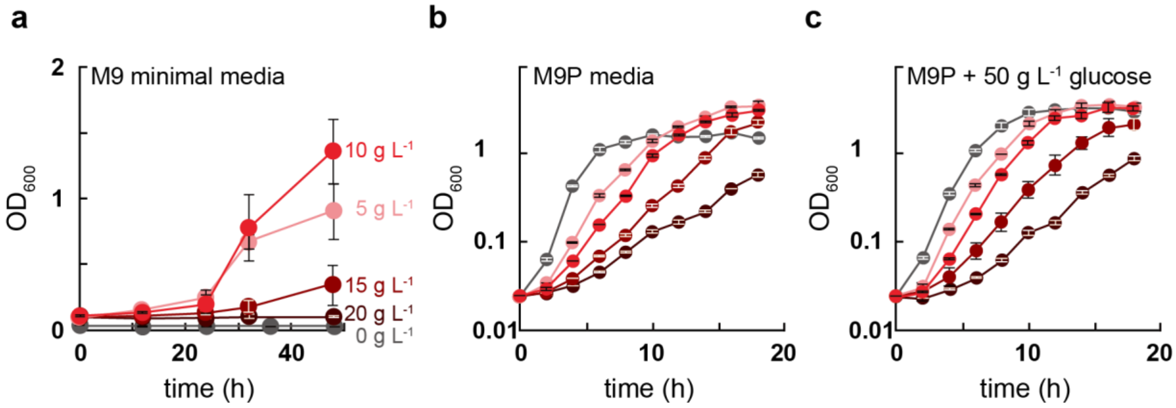

Supplementary Figure 1 Acetate toxicity test on *E. coli* growth

(**a**) Strain 1 (**Table 1**) was grown in M9 minimal media with varying concentrations of acetate as a sole carbon source. (**b** and **c**) Growth of Strain 7 (**Table 1**) in M9P media with varying concentrations of acetate (**b**) or with both 50 g L^{-1} glucose and varying concentrations of acetate (**c**). Error bars indicate s. d. (n=3).

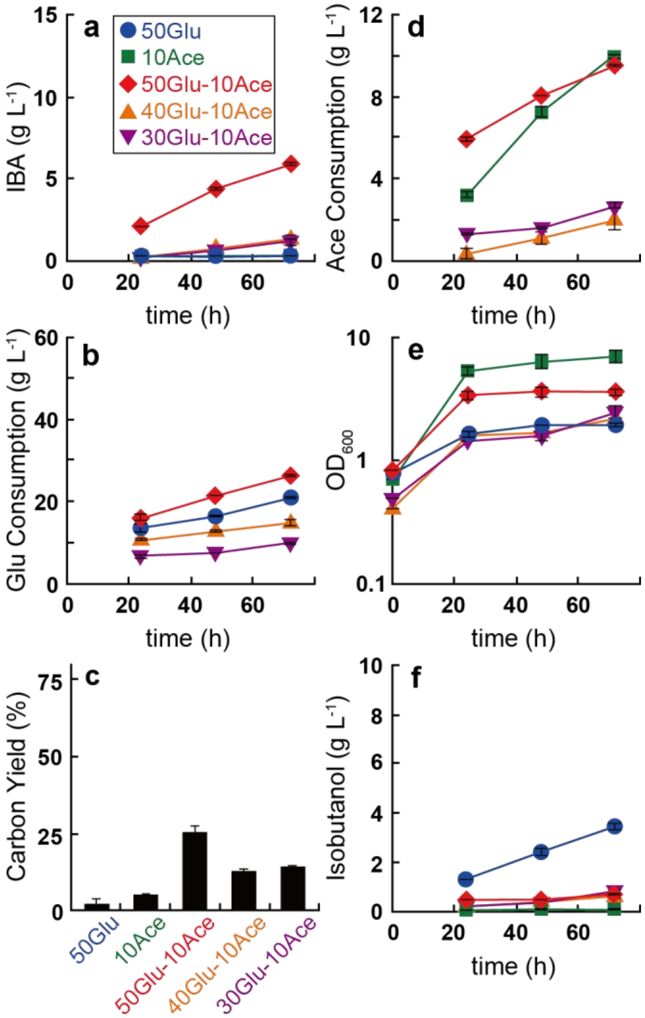

Supplementary Figure 2 IBA production with glucose and acetate in Strain 8

Strain 8 (AL2045 harboring IBA production and acetate assimilating pathways, **Table 1**) was grown in M9P media with 50 g L^{-1} glucose (50Glu), 10 g L^{-1} acetate (10Ace), or both (50Glu-10Ace, 40 g L^{-1} glucose and 10 g L^{-1} acetate (40Glu-10Ace), or 30 g L^{-1} glucose and 10 g L^{-1} acetate (30Glu-10Ace). IBA concentration (**a**), consumed glucose (**b**), consumed acetate (**d**), cell density (**e**), and isobutanol concentration (**f**) were monitored during the experiment. Carbon yield of IBA was calculated at 72 h (**c**). Error bars indicate s. d. (n=3).

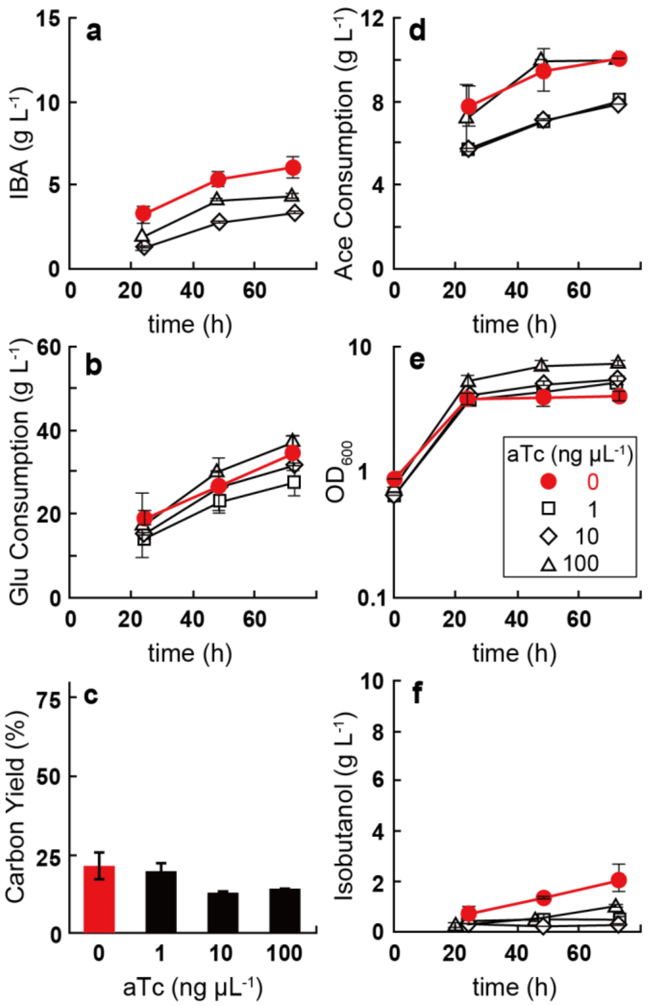

Supplementary Figure 3 Effect of expression level of *ackA* and *pta* on IBA production in Strain 9

Strain 9 (AL2045 harboring the isobutanol pathway and AckA-Pta pathway where *ackA* and *pta* are under the control of $P_L$tetO_1, **Table 1**) was grown in M9P (50 g L^{-1} glucose and 10 g L^{-1} acetate) with different concentration of aTc (0, 1, 10, or 100 ng μL^{-1}). IBA concentration (**a**), consumed glucose (**b**), consumed acetate (**d**), cell density (**e**), and isobutanol concentration (**f**) were monitored. Carbon yield of IBA was calculated at 72 h (**c**). Error bars indicate s. d. (n=3).

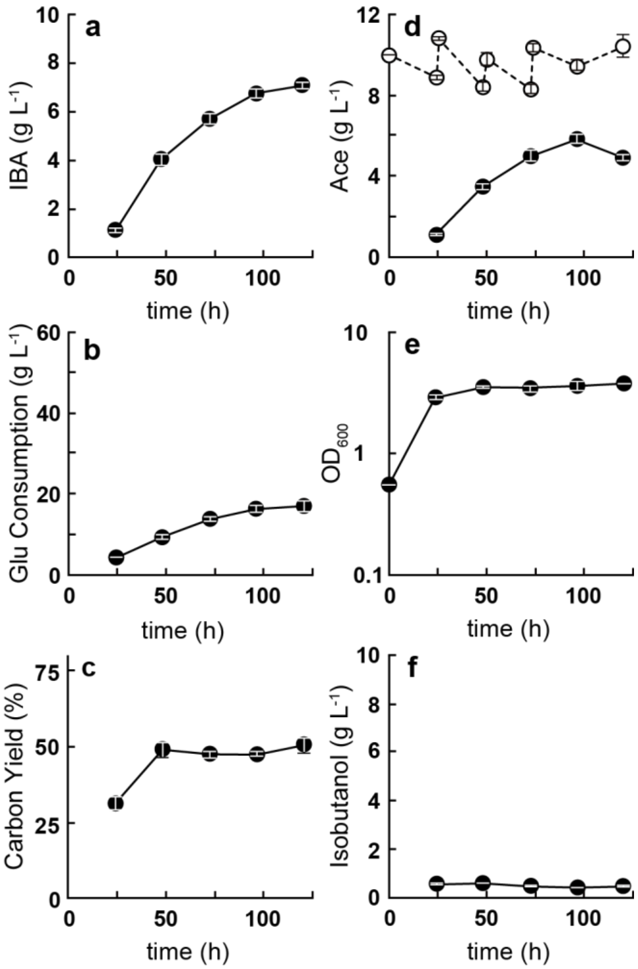

Supplementary Figure 4   IBA production with acetate-feeding in Strain 8

Strain 8 (AL2045 harboring the isobutanol pathway and AckA-Pta pathway, **Table 1**) grown in M9P media with 50 g L^{-1} glucose and 10 g L^{-1} acetate, where acetate was fed daily. IBA concentration (**a**), consumed glucose (**b**), consumed acetate (**d**), cell density (**e**), and isobutanol concentration (**f**) were monitored during the experiment. Carbon yield of IBA was calculated during the entire experiment (**c**). Open circle and closed circle indicate acetate concentration in culture and of consumed acetate in (**d**), respectively. Error bars indicate s. d. (n=3).

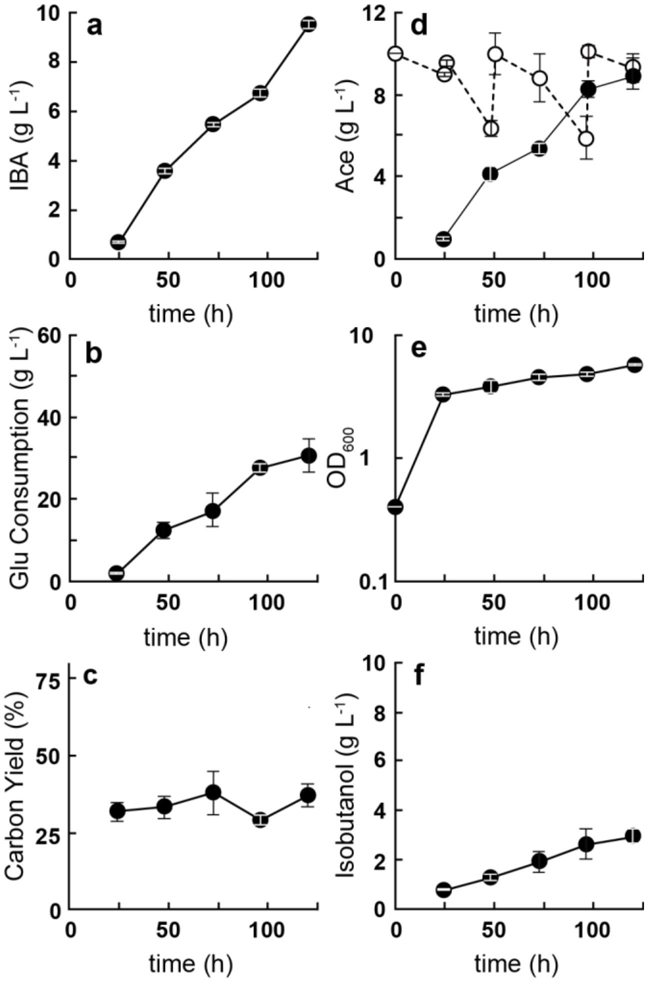

Supplementary Figure 5   IBA production with acetate-feeding in Strain 9

Strain 9 (**Table 1**) was grown in M9P media with 50 g L^{-1} glucose and 10 g L^{-1} acetate, where acetate was fed daily. IBA concentration (**a**), consumed glucose (**b**), consumed acetate (**d**), cell density (**e**), and isobutanol concentration (**f**) were monitored during the experiment. Carbon yield of IBA was calculated during the entire experiment (**c**). Open circle and closed circle indicate acetate concentration in culture and of consumed acetate in (**d**), respectively. Error bars indicate s. d. (n=3).

Supplementary Table 1 Genotype of *E. coli* host strains and plasmids in this study

***E. coli* strain**	**Genotype**	**Ref**
BW25113	*rrnB*T14 Δ*lacZ*WJ16 *hsdR514* Δ*araBAD*AH33 Δ*rhaBAD*LD78	1
JCL16	BW25113/F' [*traD36 proAB*+ *lacI*qZΔM15 (Tet^R)]	2
JCL260	Same as JCL16 but with Δ*adhE* Δ*frd-ldhA* Δ*pta* Δ*pflB* Δ*fnr*	3
AL2045	Same as JCL260 but with Δ*aceEF*	This study
**Plasmid name**	**Genotype**	**Ref**
pAL603	ColE1 ori, Amp^R, $P_L$lacO_1: *alsS-ilvCD*, $P_L$lacO1: *kivd-adhA*	4
pAL685	p15A ori, Kan^R, $P_L$lacO_1: *ATF1*	4
pAL991	p15A ori, $Spec^R$, $P_L$lacO_1: *ATF1*	This study
pAL953	Cola ori, Kan^R, $P_L$lacO_1*: ackA-pta*	This study
pAL954	Cola ori, Kan^R, $P_L$lacO_1*: acs*	This study
pAL955	Cola ori, Kan^R, $P_L$lacO_1: *mhpF-aldB*	This study
pAL956	Cola ori, Kan^R, $P_L$lacO_1: *sfGFP*	This study
pAL925	Cola ori, Kan^R, $P_L$tetO_1: *luc*	This study
pAL1022	Cola ori, Kan^R, $P_L$tetO_1*: ackA-pta*	This study

Supplementary Table 2 Oligonucleotides used in this study

name	DNA sequence
GR793	TTAAAGAGGAGAAAGGTACAATGAGTAAGCGTAAAGTCGCCATTATC
GR794	GTGACCTTTCTCCTGCATGCTCATGCCGCTTCTCCTGCCTT
SD62	GGCCCTTTCGTCTTCACCTCGAG
YT040	CATGGTACCTTTCTCCTCTTTAATGAATTCGGTCA
YT072	CTCGAGGTGAAGACGAAAGGGCC
YT092	CTACTCAGGAGAGCGTTCAC
YT101	GCTTCCCAACCTTACCAGAG
YT157	CATTAAAGAGGAGAAAGGTACCATGGGTCATCACCACCA
YT195	ATTTGATGCCTCTAGCACGCGTTTATTTGTACAGTTCGTCCATGC
YT297	TAAACGCGTGCTAGAGGCATCAAATAAAACGA
YT525	CATTAAAGAGGAGAAAGGTACCATGAGCCAAATTCACAAACACACC
YT526	ATTTGATGCCTCTAGCACGCGTTTACGATGGCATCGCGATAGC
YT634	GACTCCTGTTGATAGATCCAGTAATG
YT635	GCGCCCTCTGGTAAGGTTGGGAAGCAGCTCTCGGGTAACATCAAGGCC
YT636	ATTACTGGATCTATCAACAGGAGTCAGATTTTAATGCGGATGTTGCGATTAC
YT699	CATTAAAGAGGAGAAAGGTACCATGTCGAGTAAGTTAGTACTGGTTCTGAACTGC
YT700	ATTTGATGCCTCTAGCACGCGTTTACTGCTGCTGTGCAGACTGAATC
YT701	AAGCATGCAGGAGAAAGGTCACATGACCAATAATCCCCCTTCAGCAC
YT702	ATTTGATGCCTCTAGCACGCGTTTAGAACAGCCCCAACGGTTTATCC
YT706	GAAAACTCAACGTTATTAGATAGATAAGGAATAACCCATGATTCCGGGGATCCGTCGACC
YT707	GTTGGGCCGGCTCTTTTACTTACATCACCAGACGGCGAATTGTAGGCTGGAGCTGCTTCG
YT712	GTCTTATTGAGCTTTCCGGCGAGAG
YT713	TAATACCCTAACCACCACCGGGTCG

Supplementary Table 3 Plasmid construction by SLIC

	Vector			Insert			
Plasmid	Primer 1	Primer 2	Template	Primer 1	Primer 2	Template	Inserted Gene
pAL991	YT634	YT101	pAL685 [4]	YT635	YT636	pAL150 (Ref 5)	*Spec*^{R}
pAL953	YT040	YT297	pIM8 [6]	YT699	YT700	gDNA of *E. coli**	*ackA-pta*
pAL954	YT040	YT297	pIM8	YT525	YT526	gDNA of *E. coli*	*acs*
pAL1037	YT040	YT297	pIM8	YT701	YT702	gDNA of *E. coli*	*aldB*
				GR793	GR794	gDNA of *E. coli*	*mhpF*
pAL956	YT040	YT297	pIM8	YT157	YT195	pTrc-sfGFP	*sfGFP*
pAL925	YT072	YT054	pIM8	YT092	SD62	pZA31-*luc* (Ref 7)	$P_L$tetO$_1$: *luc*
pAL1022	YT040	YT297	pAL925	YT699	YT700	gDNA of *E. coli*	*ackA-pta*

*MG1655 (Ref 8)

Supplementary References

1. Datsenko, K.A. & Wanner, B.L. One-step inactivation of chromosomal genes in *Escherichia coli* K-12 using PCR products. *Proc Natl Acad Sci U S A* **97**, 6640-6645 (2000).
2. Atsumi, S. et al. Metabolic engineering of *Escherichia coli* for 1-butanol production. *Metab Eng* **10**, 305-311 (2008).
3. Atsumi, S., Hanai, T. & Liao, J.C. Non-fermentative pathways for synthesis of branched-chain higher alcohols as biofuels. *Nature* **451**, 86-89 (2008).
4. Rodriguez, G.M., Tashiro, Y. & Atsumi, S. Expanding ester biosynthesis in *Escherichia coli*. *Nat Chem Biol* **10**, 259-265 (2014).
5. Atsumi, S., Higashide, W. & Liao, J.C. Direct photosynthetic recycling of carbon dioxide to isobutyraldehyde. *Nat Biotechnol* **27**, 1177-1180 (2009).
6. Shen, C.R. et al. Driving forces enable high-titer anaerobic 1-butanol synthesis in *Escherichia coli*. *Appl. Environ. Microbiol.* **77**, 2905-2915 (2011).
7. Lutz, R. & Bujard, H. Independent and tight regulation of transcriptional units in *Escherichia coli* via the LacR/O, the TetR/O and AraC/I1-I2 regulatory elements. *Nucleic. Acids. Res.* **25**, 1203-1210 (1997).
8. Blattner, F.R. et al. The complete genome sequence of *Escherichia coli* K-12. *Science* **277**, 1453-1462 (1997).
